# Effects of Longitudinal Whole-Body Electromyostimulation on Maximum Strength and Power in Sportspeople and Athletes—A Systematic Review and Meta-Analysis

**DOI:** 10.3390/muscles5030049

**Published:** 2026-07-08

**Authors:** Franziska Adams, Matthias Kohl, Simon von Stengel, Andre Filipovic, Michael Uder, Wolfgang Kemmler

**Affiliations:** 1 Institute of Radiology, University Hospital Erlangen, 91054 Erlangen, Germany; franziska.hesse@fau.de (F.A.); simon.von.stengel@fau.de (S.v.S.); michael.uder@uk-erlangen.de (M.U.); 2 Faculty III: Health, Medical and Life Sciences, Black Forest University Furtwangen, 78054 Schwenningen, Germany; matthias.kohl@hs-furtwangen.de; 3 Sportclub, Verein für Bewegungsspiele (VfB) Stuttgart, 70329 Stuttgart, Germany; andre.filipovic@gmx.net

**Keywords:** whole-body electromyostimulation, sportspeople, athletes, performance, maximum strength, maximum power

## Abstract

Background: Given its practical advantages in terms of time efficiency, joint compatibility and extensive adaptability, whole-body electromyostimulation (WB-EMS) has gained considerable attention as a training method among athletes and sportspeople across numerous disciplines. The present systematic review and meta-analysis aimed to determine the impact of WB-EMS on maximum hip/lower-extremity strength and power. Methods: A systematic search of five literature databases was carried out up to 30 March 2025 in accordance with the PRISMA scheme. Effect sizes (SMD) and 95–confidence intervals (95–CI) were derived using a random-effect model incorporating the inverse heterogeneity approach. Results: Nineteen WB-EMS and 21 control groups were included. Overall, we observed favorable effects of this novel training technology on maximum strength (18 trials, SMD: 0.62, 95–CI: 0.08 to 1.16, I^2^ = 86%)) and power (8 trials, SMD: 0.38, 95–CI: 0.06 to 0.70, I^2^ = 15%). Subgroup analyses examining superimposed WB-EMS relative to voluntary exercise alone yielded low to moderate, yet statistically non-significant, effects on maximum strength (12 trials, 0.44, −0.20 to 1.08, I^2^ = 85%) and power (7 trials, 0.39, −0.02 to 0.81, I^2^ = 35%). Studies addressing the comparison of superimposed or non-superimposed WB-EMS against traditional dynamic resistance exercise reported largely equivalent outcomes for both strength and power development. Conclusion: Contingent upon the mode of application (superimposed, additional or optional), WB-EMS may exert a positive influence on maximum strength and power in sportspeople and athletes.

## 1. Introduction

Owing to its efficiency, joint-sparing properties, and high adaptability [1,2], whole-body electrostimulation (WB-EMS) represents a training technology capable of simultaneously activating all major muscle groups at variable intensities per region, rendering it attractive to sportspeople and athletes from numerous disciplines [3]. It is therefore unsurprising that most studies in this field addresses outcomes related to physical performance [3]. Due to the resistance-type character of traditional WB-EMS application (i.e., 20–30 min of intermitted, intense stimulation [1,2]), most WB-EMS trials address maximum strength and power as core study outcomes [3]. Undoubtedly, improving strength and power is crucial for most sports. Even outside traditional strength-based sports, strength and explosive power are essential components for performance, injury prevention, and efficiency in endurance and team sports [4,5]. In fact, even very early studies (e.g., [6,7]) using WB-EMS technology have already demonstrated positive effects on strength and power outcomes, as well as on study endpoints associated with these parameters. However, reliably summarizing the impact of whole-body EMS (WB-EMS) on maximum strength and power is a daunting task. This predominately refers to aspects related to specifications in WB-EMS application but also to characteristics of the corresponding comparators, i.e., the control group(s) of the trials. In this context, it is essential to understand and take into account the specific characteristics of “superimposed” WB-EMS, which is primarily applied in athletic cohorts [3], in order to reliably interpret results of WB-EMS trials. Briefly, unlike non-superimposed protocols that focus on electrical stimulation without relevant voluntary exercise, in superimposed WB-EMS programs the volitional (predominately sport-specific) exercise is overlaid by electrical stimulation. In the case of WB-EMS this encompasses all the main muscle groups and may also include supramaximal contraction. Unfortunately, rarely reported in sufficient detail, most superimposed WB-EMS studies focus on intense voluntary exercise superimposed by electrical stimulation with impulse intensities that are just sufficient to permit adequate execution of the movement (e.g., [8]). This aspect indicates that effects reported for superimposed WB-EMS cannot be solely attributed to the WB-EMS application. Indeed, depending on the rate of volitional intensity and overlying electrical current, a relevant proportion of the effect has to be attributed to the superimposed exercise. While the majority of such trials compared their WB-EMS protocol with control groups which conducted identical volitional exercise albeit without WB-EMS (e.g., [8,9,10]), others that considered superimposed WB-EMS as a whole implemented either active control groups (e.g., dynamic resistance exercise [11]) or control groups with ongoing exercise routine without any new interventions (e.g., [12]). It can clearly be expected that the effects of studies comparing superimposed WB-EMS against the underlying volitional exercise may be modest, attributable in part to the restricted sample sizes typically observed in research involving sportspeople and athletes [3]. Although not limited to WB-EMS trials, another problem for summarizing and quantifying WB-EMS effects is the large variety of performance outcomes related to maximum strength and power. Nevertheless, the work sought to deliver a comprehensive synthesis of WB-EMS effects on maximum strength and power in sportspeople and athletes. Further, we intended to reliably quantify the effect of WB-EMS in different categories according to type of WB-EMS (i.e., superimposed or not) and the exercise modalities of the respective control groups (i.e., identical volitional exercise, a newly introduced alternative intervention, or continuation of the habitual training routine). Our main hypotheses are that WB-EMS application generates significant effects on (1a) maximum strength and (1b) power; however, in detail the effect will vary considerably depending on the comparator. In this context we tested the following secondary hypotheses. 

Hypothesis 2: Superimposed WB-EMS compared to superimposed volitional exercise alone provided significantly higher effects on maximum strength (hypothesis 2a) and power (hypothesis 2b). 

Hypothesis 3: Superimposed WB-EMS compared to ongoing training routine without new intervention provided significantly higher effects on maximum strength (hypothesis 3a) and power (hypothesis 3b). 

Hypothesis 4: Non-superimposed WB-EMS compared to ongoing training routine without new intervention provided significantly higher effects on maximum strength (hypothesis 4a) and power hypothesis (4b). 

Hypothesis 5: The effect of superimposed or non-superimposed WB-EMS on maximum strength (5a) and power (5b) will not be superior compared with newly added resistance-type exercise.

## 2. Results

**Figure 1:**
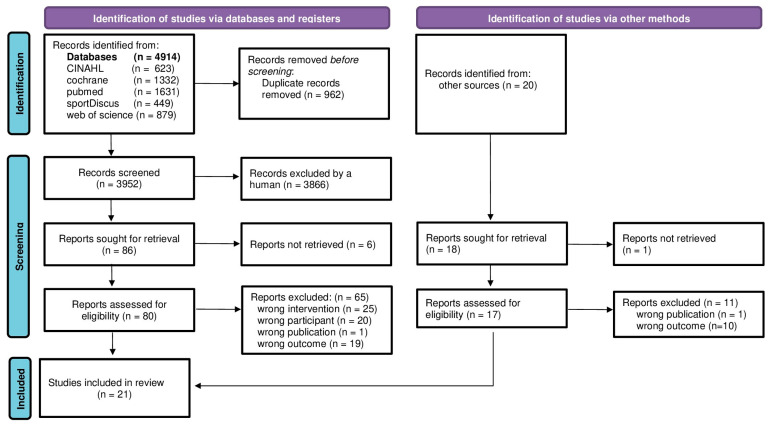
Flow chart of the present systematic literature search according to PRISMA [29].

Finally, the 4914 records are as summarized: 18 projects published in 21 longitudinal controlled studies [8,9,10,11,12,13,14,15,16,17,18,19,20,21,22,23,24,25,26,27,28] with 19 WB-EMS groups (248 participants) and 21 control groups (292 participants) (Figure 1).

### 2.1. Publication and Study Characteristics

Table 1 shows the publication and study characteristics of the eligible studies. Aside from two trials that were not randomized [17,19], all remaining studies followed a randomized controlled trial including one study [25] that applied a randomized cross-over design. Studies were published between 2015 and 2022; half of the projects (9 of 18) were conducted in Germany [8,9,10,13,14,19,21,22,25,28]. The majority of studies compared one WB-EMS group with one control group. One project [11] implemented two WB-EMS groups; in other trials, WB-EMS was compared with two distinct control groups [12,13,14,15,16,24]. Study group sizes ranged from n = 5 [17] to n = 27 [28] subjects.

### 2.2. Participant Characteristics

Table 1 summarizes the characteristics of the participants. In summary, five projects (6 comparisons) included hobby sportspeople [12,23,24,27,28], ten projects (12 comparisons) focused on advanced sportspeople [9,11,15,16,18,19,20,21,22,25] including physical education/sport students (n = 6) and three projects (four comparisons) included semi-professional or professional athletes [8,13,14,17,30]. Participants of eleven projects (14 comparisons) can be considered as allrounders [9,10,11,12,18,20,21,22,23,24,26,27,28], six projects (8 comparisons) focused on team sport [8,13,14,15,16,17,19,25] and one project included golf players [28] categorized as “precision sport”. Ten projects included only male, four only female and four included mixed cohorts (Table 1). Apart from one study that included male hobby golfers aged 18–70 years old [28] and one study that focused on younger men aged 15–20 years [30], participants had a mean age of 20–30 years. One trial focused on the rehabilitation of athletes with injured hip joint connective muscles [17]; all the other projects included healthy sportspeople and athletes. Further, in only one study cohorts were overweight [28], at least when applying a BMI-based cut-off (i.e., >25 kg/m^2^) (Table 1).

### 2.3. Exercise Characteristics

Of primary importance, most of the projects (n = 14, Table 2) applied superimposed WB-EMS protocols [8,9,10,11,12,13,14,15,16,18,19,20,21,22,25,26,27]. Basic volitional exercise superimposed by WB-EMS included power and/or dynamic isometric and/or isometric resistance exercises, jumping or cycling (Table 2). The remaining studies that focused on non-superimposed WB-EMS [17,23,24,28] conducted easy movements/exercises during the impulse phases which per se should not affect performance outcomes in well-trained cohorts.

Study duration varied from 4 [9,21] to 16 weeks [28]. Weekly WB-EMS volume differed substantially, ranging from 13 min (1 × 13 min [20]) to 3.5 h (3.5 × 60 min [21]); however, most trials implemented programs compromising 1–2 weekly sessions of 9–30 min [8,9,10,11,12,13,14,18,19,20,22,24,25,26,27,28]. All the trials applied bipolar (biphasic) stimulation currents [1] (Table 2). In detail, with the exception of two studies that applied slightly higher stimulus frequencies (100 Hz [18], 120 Hz [20]), all the projects were stimulated with 80–90 Hz and an impulse breadth of 300–400 µs. Continuous WB-EMS application, i.e., stimulation over the entire session, was only applied by Evangelista et al. [12]. All the remaining projects used protocols with brief stimulation bouts (typically during the volitional exercise) interspersed with short impulse breaks (Table 2). Of note, in non-superimposed projects “exercise intensity” focused predominately on WB-EMS stimulus intensity. The few studies to focus on this approach (see above) scheduled impulse intensities of 6 (hard+) to 8 (very hard+) when using the Borg CR10 rating scale. In contrast, in studies that focused on superimposed WB-EMS (see above) exercise intensity refers to the product of voluntary exercise and adjuvant WB-EMS together. While difficult to quantify precisely (e.g., “60–100% device capacity”), it can be concluded that all trials applied at least moderate to high, and in some cases very high (e.g., “maximum tolerable intensity”), exercise intensity (Table 2).

### 2.4. Control Group Characteristics

Of the 22 control groups (one study [11] compared two WB-EMS interventions with one CG), 13 performed the identical underlying exercises as the (superimposed) WB-EMS groups (i.e., power and/or resistance exercises: n = 9, jumping: n = 3, cycling: n = 1) (Table 2). One project compared isometric exercises superimposed by WB-EMS with a DRT-control group. Three superimposed WB-EMS projects further compared their WB-EMS groups with CGs with an ongoing training routine [12,13,14,16]. From the five CG of the non-supervised WB-EMS groups, three applied an ongoing training routine [17,24,28] and two [23,24] implemented a DRT-program (Table 2).

### 2.5. Unintended Side Effects, Lost to Follow-Up, Adherence (WB-EMS Group)

Unfortunately, five projects failed to report WB-EMS-related unintended side effects and/or did not reply to our requests for clarification (Table 2). Of the remaining 13 projects, no study documented adverse or unintended side effects attributable to the WB-EMS application. Two team sport projects [8,19], however, reported increased creatine-kinase (CK) levels 24 h and 72 h after superimposed WB-EMS. This phenomenon is frequently reported after WB-EMS application with high stimulus intensity during the early conditioning phase [31], predominately in “high responders” (i.e., [32]). Lost to follow-up, these were provided by 14 studies that ranged between 0% [8,10,11,15,16,19,22,26] and 33% [28]. The exceptionally high withdrawal rate in the latter study [28] was related to the very stringent COVID-19 induced lock-down in Bavaria (Germany). The attendance rate across the 13 projects that reported this parameter consistently approached 100%, facilitated by the option to compensate for missed WB-EMS sessions.

### 2.6. Methodologic Quality

Methodologic quality according to PEDro [33] is presented in Table 1. Overall, the methodological quality scores varied from 3 to 8 (maximum of 10 points) and may therefore be classified as low to moderate [34]. The key factors for this unsatisfying finding were related to the PEDro dimension of “allocation concealment”, “blinding of participants” and/or “blinding of therapists”. However, given that the latter two aspects are not reliably applicable to WB-EMS trials (or exercise studies in general), a score of 8 points should be considered a realistic ceiling. Indeed, the TESTEX Score [35] that did not consider “blinding of participants” and/or “blinding of therapists” and awarded points for exercise-specific criteria generated (on average) better results (i.e., 7 to 14 out of 15 score points) for the individual studies. However, it should be noted that we have taken into account the feedback from the authors contacted regarding adherence, attendance, and adverse effects in the evaluation.

### 2.7. Study Outcomes

All trials assessed outcomes related to maximal strength performance (Table 3). With the exception of two studies that focused on maximum handgrip [20] and arm flexor/extensor strength [23], all the other projects determined maximum hip/lower-extremity strength by leg press, leg extension/flexion, squat exercises or [17] hip adduction [17] (Table 3). Maximum strength assessments included 1RM-tests, repetition to fatigue tests with 1RM prediction equations, isometric tests (Fmax) and/or isokinetic tests. Of note, in projects that reported multiple lower-extremity outcomes (e.g., leg press, leg extension, flexion; e.g., [9]), the leg press result was selected for inclusion in the meta-analysis.

**Figure 2:**
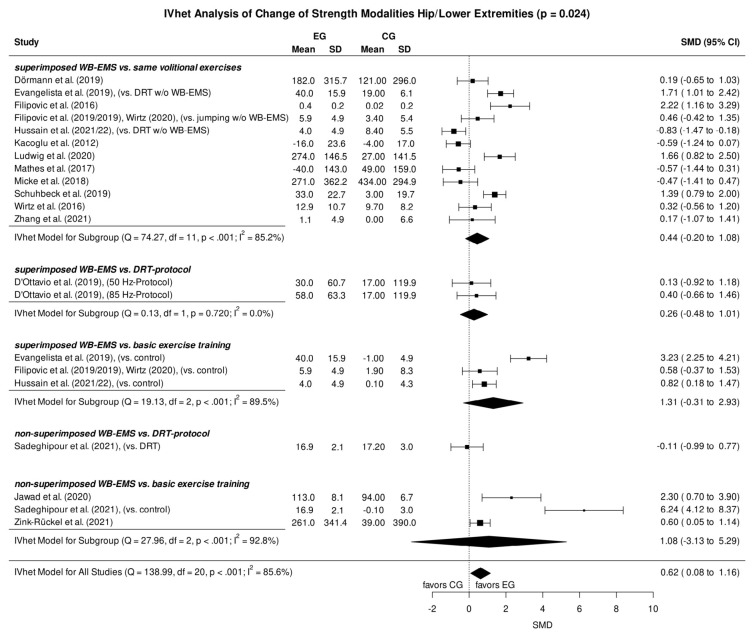
Forest plot of WB-EMS effects on hip/lower-extremity strength [8,9,10,11,12,13,14,15,16,17,18,19,21,22,24,25,26,27,28].

Maximum upper body/extremity strength was determined by five projects with seven comparisons [11,16,20,23,27]. Five comparisons focused on the bench press exercise, one study each addressed maximum handgrip [20] and arm flexor/extensor strength [23]. Of note, for reasons of consistency only comparisons that focused on the bench press exercise were included in the quantitative analysis (Figure 2). In this context Martin-Simon et al. [20] reported comparable (vs. CG) but significant increases for handgrip strength after superimposed WB-EMS. In parallel, the study of Qin et al. [23] which compared non-superimposed WB-EMS vs. conventional DRT (Table 2) reported significantly higher Fmax increases in the elbow flexors and extensors in favor of the WB-EMS group.

Eight projects [9,10,11,21,22,25,27] with nine comparisons additionally determined outcomes related to maximum power of the lower extremities. Assessments included squatting [11,27], leg press [9,10,22], leg extension [21] and vertical jump power [20,25] (Table 3, Figure 2).

### 2.8. Meta-Analysis Results

#### 2.8.1. Hip/Lower-Extremity Maximum Strength Outcomes

Figure 2 displays the impact of WB-EMS on maximum strength of the lower extremities as determined by leg press, knee extension, squat or hip adduction assessments. Overall, regardless of the imputation strategy (mean, minimum, maximum SD), the IVHet model showed a significant moderate effect (*p* = 0.024; SMD: 0.62, 95% CI: 0.08 to 1.16), with considerable heterogeneity across studies (I^2^ = 85.6%). More surprisingly, we also observed a high degree of heterogeneity within the subcategories. Additionally, apart from the research issue of superimposed WB-EMS versus underlying volitional exercise, the sample size/statistical power (2–3 studies each) of all the other subgroup analyses was very limited; thus, reliable and meaningful effects cannot be necessarily expected. Most revealingly, due to the large heterogeneity within the few studies, the meta-analytic approach that addressed the issue of non-superimposed WB-EMS versus basic exercise group was non-significant although all the underlying studies reported significant effects (Figure 2). Addressing the results in more detail, we observed a small (SMD: 0.44), non-significant effect (*p* = 0.18) for the scenario “volitional exercise with and without WB-EMS” (Figure 2, upper graph). Less reliable due to limited statistical power (one study with two WB-EMS groups only) but, however, expected, we observed a missing effect of superimposed WB-EMS versus traditional DRT (*p* = 0.49). This result was confirmed by the result of the “non-superimposed WB-EMS versus conventional DRT” research issue (*p* = 0.80), that includes only one study [24]. Although to be treated with caution, we did not observe significant effects for the research issue that compared superimposed (*p* = 0.067) or, as mentioned above, non-superimposed (*p* = 0.62) WB-EMS protocols versus control groups which maintained their habitual exercise programs (Table 2).

In summary, we confirmed our main hypothesis (1a) of significantly higher effects of WB-EMS on maximum strength of the lower extremities compared to control. However, the more detailed hypothesis that addresses the significant superiority of superimposed WB-EMS protocols versus the underlying volitional exercises (2a) had to be rejected. Under the premise that the limited number of trials included in the subgroup analyses (n = 1–3) prevents reliable analyses, we refrained from the formal confirmation of rejection of hypotheses 3–5.

#### 2.8.2. Maximum Strength Outcomes Bench Press

**Figure 3:**
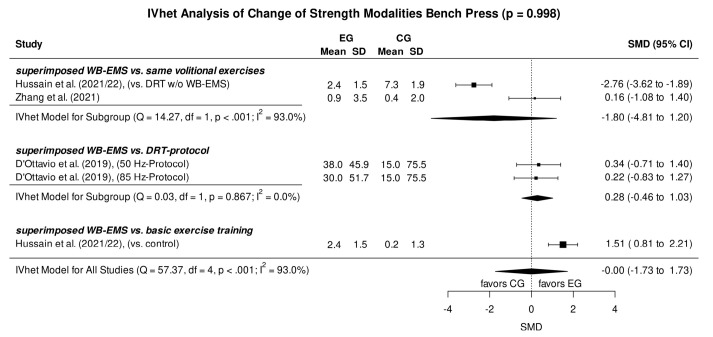
Forest plot of WB-EMS effects on upper-extremity strength (bench press exercise) [11,15,16,27].

Summarizing the effect of (superimposed) WB-EMS protocols (Figure 3, n = 5), we did not observe positive effects on maximum bench press strength (SMD = 0.00, −1.73 to 1.73), independent of the imputation strategy. In parallel to lower-extremity maximum muscle strength, the heterogeneity of results between the trials was considerable (I^2^: 93%). Unfortunately, the low number of studies (n = 1–2) among the subcategories prevents a reliable analysis; nevertheless, the results have been briefly presented. In detail, one of two studies that compared superimposed WB-EMS vs. the same volitional exercise only reported significant differences in favor of the control group—a result that is hard to interpret. In parallel to their results on lower-extremity strength, D’Dottavio et al. [11] observed largely similar effects of superimposed WB-EMS vs. DRT (*p* = 0.46), while the study project of Hussain et al. [15,16] reported significantly higher maximum strength effects of superimposed WB-EMS vs. ongoing training routine only (*p* < 0.001).

Due to the low numbers of studies, maximum upper body/extremity strength was not addressed by dedicated hypotheses.

#### 2.8.3. Maximum Power of the Lower Extremities

**Figure 4:**
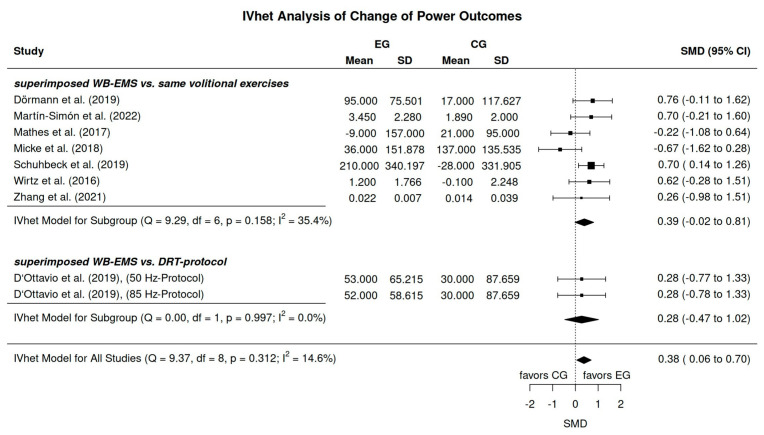
Forest plot WB-EMS effects on maximum hip/lower-extremity power [9,10,11,21,22,25,27].

Summarizing the results of the studies, we determined a significant (0.021) but low effect (SMD: 0.38) of superimposed WB-EMS versus control on maximum power. Subgroup analyses (imputation minimum/maximum SD) confirmed this result. In contrast to maximum strength data, heterogeneity between the results of the trials included in the analysis was low (I^2^ = 14.6%). However, studies included in the analysis addressed only two research issues Figure 4), one of them (superimposed WB-EMS vs. DRT) with insufficient statistical power. In detail, all studies focused on superimposed WB-EMS programs compared with either the identical underlying volitional exercise or a conventional DRT protocol [11]. We determined non-significant (*p* = 0.061) effects (SMD: 0.39) of superimposed WB-EMS vs. the same volitional exercise only and no relevant differences (*p* = 0.47) between the two superimposed WB-EMS groups of D’Ottavio et al. [11] versus their corresponding DRT-control group (Figure 4).

In parallel to maximum strength of the hip/lower extremities, we verified the general effectiveness of WB-EMS compared to control (hypothesis 1b). Although borderline, we have to reject hypothesis 2a, which postulated the superiority of the WB-EMS application over its superimposed exercise counterpart. Unfortunately, the lack of studies prevented a reliable decision on the other hypotheses (3b, 4b, 5).

### 2.9. Publication/Small-Study Bias

**Figure 5:**
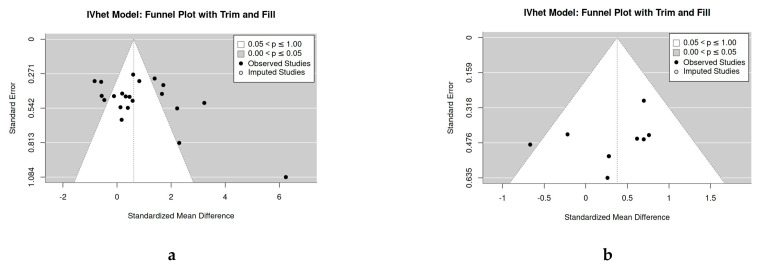
Funnel plots of results for lower-extremity strength (**a**) and power (**b**).

Of note for the detection of publication/small-study bias and asymmetries, we included all the studies regardless of the category. The funnel plot with trim-and-fill analysis suggests no evidence for a publication/small-study bias (Figure 5a). Further, neither rank nor regression test revealed significant asymmetry. Ultimately, the LFK index (1.07) showed only slight asymmetry.

Similarly, the funnel plot, rank test (*p* = 0.36), and regression test (*p* = 0.73) do not provide evidence of publication or small-study bias in the trials included in the analysis of maximal upper-body strength (i.e., bench press). The LFK index (−0.99) confirmed the finding of minor asymmetry. However, due to the limited number of studies this result should be interpreted with caution.

Figure 5b illustrates the lack of relevant asymmetry for studies included in the analysis for maximum power. This result was in line with the results of the rank (*p* = 0.36) and regression test (*p* = 0.26), while the LFK index (−2.35) and corresponding DOI plot on the other hand suggest major asymmetry related to a potential publication/small-study bias.

## 3. Discussion

This systematic review and meta-analysis constitute the first work to systematically synthesize and quantify the impact of WB-EMS on maximum strength and power outcomes in sportspeople across varying performance levels. In summary, our main meta-analysis on maximum strength and power of the hip/leg extensors revealed significant effects of WB-EMS versus control groups on both outcomes (Figure 2 and Figure 4, upper graph). In general, improving the strength and power of these muscle groups is important for most sports. It forms the foundation for explosive movements, changes in direction, jumps, sprints, and force transmission, as well as for injury prevention [4,36]. Nevertheless, this overarching finding provides limited guidance for sportspeople, athletes, or coaches deliberating whether to integrate WB-EMS into their training regimens.

Reviewing WB-EMS concepts, similar to the present work (Figure 2 and Figure 4, upper graphs), the time-efficient approach to superimpose already intense sport-specific exercise by WB-EMS is the most popular strategy for increasing maximum performance in sportspeople and athletes [2]. Examining our non-significant finding for maximum strength and power in greater detail, four studies addressing strength [8,12,19,25] and one study that focused on power outcomes [25] reported significantly higher effects of superimposed WB-EMS. In contrast, two studies [15,16,18] listed significant or at least pronounced effects for maximum strength development in favor of the CG. Upon reviewing both studies, no discernible particularities in cohort composition, intervention design or outcome variables could be identified that might account for this unexpected finding.

In general, it is clear that the effect of WB-EMS added to intense or near-maximum voluntary exercise (compared to intense exercise alone) might be too small to generate statistical significance at least when considering the limited statistical power of our analysis. Moreover, the aspect that exercise-induced adaptations in maximum strength and power tend to be attenuated in well-trained populations (‘principle of diminishing returns’ [5]), further impairing the statistical power of the underlying studies and correspondingly the meta-analyses. However, even marginal improvements in performance indicators are of considerable importance for advanced sportspeople and athletes [37], an aspect that might justify the feasible application of superimposed WB-EMS during traditional exercise, even under the premise that positive evidence is presently limited.

Although the statistical power of the other “scenarios” (hypotheses 3–5) is very limited and did not allow reliable recommendations, we would like to briefly address these, particularly from a pragmatic point of view, important research issues. Firstly, the comparison of superimposed or non-superimposed WB-EMS programs with DRT highlights the character of WB-EMS as a safe, efficient and joint-friendly option [1] to conventional exercise. In summary, the largely equivalent outcomes of both training approaches, irrespective of the outcome variable [11,24], support the notion that WB-EMS may serve as a viable substitute for conventional DRT in the context of strength and power development. Even more important for introducing WB-EMS, the comparison of WB-EMS, be it superimposed or not versus ongoing training routine, addresses the relevance of adding WB-EMS to the current training program as a new intervention. Considering the increased effort of adding WB-EMS to training routines, there should be at least some evidence for positive effects before its introduction. Due to the fact that the subgroup analyses which address this issue (Figure 2 and Figure 4, lower graph) were considerably biased by the large heterogeneity between the trials [12,13,14,15,16,17,24,28], we feel it is appropriate to refer to the individual trial results on maximum strength (power was not addressed by corresponding trials). In summary, with six of seven comparisons reporting significant positive effects in favor of the WB-EMS intervention, the body of evidence supporting the beneficial effects of WB-EMS is compelling. Correspondingly, there is considerable evidence to recommend adding WB-EMS to the habitual exercise routine at least to increase maximum strength.

An aspect that has not yet been addressed is the use of WB-EMS for injury prevention in ambitious sportspeople and athletes. While changes in maximum strength and power have limited relevance for some disciplines, the prophylaxis of injuries and complaints that prevent the proper conduct of training and competition are of overall importance. Apart from musculoskeletal lesions, low back pain that is very common in athletes [38] can be effectively reduced by WB-EMS [39], again as an option to the much more time-consuming and less joint-friendly traditional resistance exercise training.

Some of the study characteristics and limitations made in this systematic review might be challenging for properly interpreting the findings. (1) One may argue that when addressing hip/lower-extremity strength and power it does not matter whether WB-EMS or locally applied EMS was provided. Here, we do not agree. Apart from the greater surface area of the electrode pads, which cover the entire thigh or, where intended, the calf region, WB-EMS included gluteal and trunk muscles that were either directly involved in leg press or squatting exercises or provide trunk stability. Thus, WB-EMS-induced effects on maximum strength and power should be more pronounced compared to traditional local EMS applications. (2) Borderline sufficient for the main analyses, sample size and corresponding statistical power was not always appropriate to reliably address all the research questions. We anticipated the low number of WB-EMS studies on upper body/extremity strength and power outcomes and thus did not address this domain through dedicated research issues; however, for lower-extremity strength development in particular, we expected many more studies in the different categories. (3) The present study focuses on sportspeople; unfortunately, a reliable definition of such a cohort is not available in the literature. Our aim was to include individuals for whom an increase in performance is of significant importance. In summary, this group should be considered as performance-oriented sportspeople ranging from ambitious hobby sportspeople to elite athletes. After intense discussions, we applied a minimum eligibility threshold of ≥2 sessions/week over the preceding two years for hobby sportspeople. Sport students and sportspeople participating in competitions were categorized as “advanced sportspeople”, whereas semi-professional and professional individuals were classified as “athletes” [40]. Consequently, the study cohort cannot be regarded as homogeneous, although a shared characteristic is the high relevance attributed to changes in performance outcomes. Stratified analyses by training status, age group, or sport category might have improved interpretability of our results. However, due to limited statistical power we refrained from dedicated subgroup analyses on these issues. (4) Apart from the differences in WB-EMS protocols, assessments of strength and power vary considerably between the studies (Table 3). This refers not only to the exercise (squat, leg press, leg extension) but also to the mode of contraction (i.e., isometric, isokinetic, dynamic) which further complicates drawing a clear conclusion about the efficacy of WB-EMS in the strength and power domain. (5) We did not conduct a joint analysis of different regions (i.e., lower and upper extremities) to derive more detailed results. However, our approach of subsuming maximum squat, leg press, leg extension and adduction performance under “maximum hip-/lower extremity strength” is open to questioning. This also refers to the aspect that we did not separate for different modes of assessments (i.e., isometric, isokinetic, 1 RM, Table 3), which also prevents replacement of the SMD by more familiar units. In parallel to (3), separate (sensitivity) analyses by test types might have improved interpretability (and reduced heterogeneity between the trial results); however, the statistical power was too limited to address these issues. (6) Maximum power data was consistently reported for the hip/lower extremities only. Of note, although in general jumping performance can be subsumed under “power”, we included results on jumping performance only when reported in “Watt”. (7) The PEDro [33] and TESTEX scale [35], both particularly dedicated to physiotherapy and exercise studies, was used to determine the methodologic quality of the trials. However, both tools are not perfectly applicable for randomized cross-over trials and in particular non-randomized trials (Table 1). (8) From a scientific point of view, it would be helpful if the WB-EMS protocols used a (more) standardized application to draw reliable conclusions and to allow more meaningful quantitative analyses. On the other hand, standardized protocols might be suboptimal since training regimens should be tailored as closely as possible to the athlete’s current fitness level or/and the specific demands of the type of exercise. (8) The extent to which the present findings are generalizable to other populations (i.e., external validity), in particular non-athletic cohorts, remains uncertain. Most importantly, WB-EMS combined with high-intensity voluntary exercise is less commonly used in scientific and commercial WB-EMS protocols designed for the general population [1,2]. Accordingly, results derived predominantly from superimposed WB-EMS trials cannot be readily generalized to this population.

## 4. Materials and Methods

This article builds upon the systematic literature search of the comprehensive systematic review and evidence map by Reinhardt et al. [3], while employing more detailed eligibility criteria with respect to the outcomes of interest. The review was conducted in strict accordance with the Preferred Reporting Items for Systematic Reviews and Meta-Analyses (PRISMA) Statement and was prospectively registered in PROSPERO (CRD420250646327).

### 4.1. Eligibility Criteria

#### 4.1.1. Population

Eligible participants comprised athletes, advanced sportspeople, and recreational/hobby sportspeople who had exercised at least twice per week during the preceding two years. Advanced sportspeople were defined as competitive sportspeople and physical education/sport students, whereas semi-professional and professional sportspeople were categorized as “athletes” [40].

#### 4.1.2. Intervention

Studies were eligible if they applied WB-EMS, defined as “simultaneous application of electric stimuli via at least six current channels or participation of all major muscle groups, with a current impulse effective to trigger muscular adaptations” [1].

#### 4.1.3. Comparators

Eligibility was restricted to studies incorporating one or more active or inactive control groups. Studies evaluating and comparing multiple WB-EMS interventions without a separate non-WB-EMS control group (e.g., [41,42]) were not included.

#### 4.1.4. Outcomes

This review focuses on study endpoints related to maximum muscular strength and power. We particularly focus on exercises of the lower extremities, although outcomes related to upper body/extremity strength were also considered.

#### 4.1.5. Study Design

Eligibility was restricted to longitudinal controlled trials with either randomized or non-randomized study designs.

### 4.2. Information Sources

Five electronic databases—CINAHL (via EBSCOhost), CENTRAL, MEDLINE (via PubMed), SPORTDiscus (via EBSCOhost), and Web of Science (via Clarivate)—were searched from database inception to 6 March 2025 without language restrictions (Table A1).

### 4.3. Literature Search

A predefined search strategy was established [3] using controlled vocabulary (MeSH terms in MEDLINE and CINAHL Subject Headings in CINAHL), supplemented by relevant keywords and synonymous terms in the following search queries: WB-EMS OR “whole body electro myo stimulation” OR electromyostimulation OR “electrical muscle stimulation” OR electro-myo-stimulation OR electrostimulation OR “integral electrical stimulation” OR “whole-body electrical muscle stimulation”) AND (athletic OR athlete OR sport OR performance OR trained). Additionally, the reference lists of included articles were reviewed for further relevant studies (Table A1).

### 4.4. Selection Process

The screening of titles, abstracts, and full texts was conducted independently by three reviewers (M.P., F.A., W.K.) based on the established PICOS eligibility criteria. Disagreements arising during the selection process were addressed and resolved through consensus discussions. If necessary, the authors were contacted by email up to three times over a four-week period to clarify missing, incomplete, or ambiguous information.

### 4.5. Data Management

EndNote (Clarivate, PA, USA) was utilized to download and organize search results, as well as to conduct title, abstract, and full-text screening. Duplicate records were identified and removed in accordance with the approach outlined by Bramer et al. [43].

### 4.6. Data Extraction

Data from eligible studies were extracted by one reviewer (F.A.) using a Microsoft Excel spreadsheet, while a second reviewer (W.K.) independently verified the extracted information. Any discrepancies between the reviewers were resolved through discussion. The data extraction form was structured into five subcategories: (a) study and publication characteristics; (b) cohort and participant characteristics; (c) intervention characteristics, including detailed information on the WB-EMS protocol; (d) loss to follow-up, attendance, and harms or adverse events/effects; and (e) study endpoints. Special attention was given to the exercise protocols implemented in both the WB-EMS and the CG. which were categorized according to superimposed WB-EMS (yes or no) and exercise in the control group (i.e., identical volitional exercise compared to WB-EMS, newly other added intervention, ongoing training routine).

### 4.7. Quality Assessment

Using the Physiotherapy Evidence Database (PEDro) Scale Risk of Bias Tool [33], two independent reviewers (F.A. and W.K.) assessed studies for methodologic quality and risk of bias. In parallel we applied the TESTEX (Tool for the assEssment of Study qualiTy and reporting in Exercise) scale that further included specific exercise specific items [35].

Interrater reliability for both tools was high (Cohens Kappa ≥ 0.77) and disagreements were resolved by discussion. Studies were classified according to methodological quality as follows: <5 score points: low; 5–7 score points: moderate; and >7 score points: high [34].

### 4.8. Data Synthesis

Missing standard deviations (SD) were calculated following the methodology outlined in the recent comprehensive meta-analysis by Shojaa et al. [44]. Specifically, standard errors (SE) and confidence intervals (CI) were transformed into SDs [45]. In studies lacking any reported measure of change variability, the mean SD was imputed based on correlations between baseline and endpoint values obtained from other studies [45].

Table 1, Table 2 and Table 3 provide an overview of study, publication, cohort, participant, and intervention characteristics. The primary outcome was the change in maximum strength of the hip and lower limbs as determined by leg press (preferred), leg extension or squat exercise independently of the mode of action (i.e., isometric, dynamic or isokinetic). In parallel, upper-extremity strength results as determined by bench press exercise were also summarized in a meta-analytic approach. Lastly, maximum muscular power of the hip/lower-extremity muscles as determined by leg press, leg extension, squatting or jumping exercise was quantified. When multiple strength or power outcomes were available, preferably maximum hip/lower-extremity strength or power as determined by squatting, leg press exercises (subordinated leg extension, leg adduction) were included in the analysis. When muscular power (in W) was determined by maximum jumping performance, the counter movement jump was preferably considered (Table 3).

WB-EMS Interventions were categorized by two independent reviewers (W.K., S.v.S.) according to the mode of WB-EMS (superimposed or non-superimposed) and the type of comparator, i.e., (1) identical volitional exercise protocol but without WB-EMS, (2) active control with a newly introduced DRT exercise protocol or (3) a control group that maintained their habitual exercise protocol.

### 4.9. Statistical Analysis

Random-effects meta-analyses were conducted with R statistical software (4.5.1 patched [46]) using the metafor package [47]. Continuous data were combined using standardized mean differences (SMDs) and 95% confidence intervals (95% CIs). The primary analysis consisted of a meta-analysis employing the robust inverse variance heterogeneity (IVhet) model [48], in anticipation of substantial between-trial heterogeneity. The Cochran Q test assessed heterogeneity for the variability between studies. The degree of heterogeneity was evaluated using the I^2^ statistic, with values of 0–40% classified as low, 30–60% as moderate, 50–90% as substantial, and 75–100% as considerable heterogeneity [49]. Alongside conventional funnel plots, a regression test and rank correlation were used to assess the effect estimate and its standard error via the t-test and Kendall’s τ statistic for potential publication bias. We also performed a trim-and-fill analysis using the L0 estimator suggested by Duval et al. [50]. We further used Doi plots and the Luis Furuya-Kanamori (LFK) index [51] to examine potential asymmetry. LFK values ranging within ±1 were regarded as negligible, while values from ±1 to ±2 were classified as minor asymmetry. Values greater than ±2 were considered indicative of major asymmetry. We conducted sensitivity analyses to evaluate the robustness of the overall findings with respect to the selected imputed correlation coefficient (minimum, mean, or maximum values).

### 4.10. Sensitivity and Subgroup Analyses

Sensitivity analyses focused on the impact of different imputation strategies, specifically the use of minimum correlation (resulting in maximum SD) versus maximum correlation (resulting in minimum SD). For the primary analysis, results based on the mean of these correlations were used.

Subgroup analyses focus on comparisons of superimposed WB-EMS with non-WB-EMS control groups with (1) identical volitional exercise protocols, (2) DRT-protocols, (3) ongoing exercise without additional intervention and non-superimposed WB-EMS-protocols with (4) DRT-protocols and (5) no additional intervention. Results were considered statistically significant at *p* < 0.05. Effect sizes were classified based on SMD values, with 0.2 indicating a small effect, 0.5 a medium effect, and 0.8 a large effect.

## 5. Conclusions

Given the limited number of available studies, which differ substantially with respect to training level, whole-body EMS protocol, control group, outcome measures and methodological quality/risk of bias and particularly heterogeneity between the trials, this study can only provide limited evidence regarding the effectiveness of WB-EMS on muscle strength and power in trained cohorts.

Although a positive overall effect of WB-EMS on maximum muscle strength and power in sportspeople and athletes was observed, the effect of WB-EMS is contingent upon its mode of application (i.e., superimposed, additional or optional) and, naturally, on the type of control group employed (i.e., same or other voluntary exercise, no corresponding activity). Nevertheless, considering that even marginal performance gains are of relevance for sportspeople and particularly athletes, the statistical power of this analysis may still be inadequate to identify clinically meaningful changes. Due to the various possible applications, it is difficult to summarize the practical implications of the findings for coaches and athletes. Despite limited evidence, we would like to suggest the application of WB-EMS (1) additionally to power and strength exercises (i.e., superimposed WB-EMS) in sport games (e.g., soccer, ice hockey) and (2) as an alternative to less time-efficient resistance exercise programs in the area of musculoskeletal injury prevention. A key area of application here would be the optimized use of WB-EMS, for example, for the prevention (after screening) and treatment of back pain, muscular imbalances, or joint instabilities [52]. Nevertheless, to adequately address, in particular, the significance of superimposed WB-EMS (versus the same voluntary exercise) in advanced exercising cohorts, further well-designed WB-EMS trials with sufficient statistical power are warranted. To date, differences in study designs, methods for measuring strength and performance, and methodological limitations of the studies have limited the quality of evidence to clearly recommend WB-EMS as a means for developing strength and performance in sportspeople and athletes.

## Figures and Tables

**Table 1:** Study features and participant characteristics reported by the eligible trials.

	Study with First Author, Year	Study Design	Number of Study Arms	Number of Total Participants/Study [n]	Sex	Age [Years]	Body Mass Index (kg/m^2^) ^1^	Exercise/Sport Level	Sport Category	PEDro Score	TESTEX Score
1	Dormann et al. 2019 [9]	RCT	two	28	♀	21 ± 2	22 ± 2	Advanced	Non-specific	4	10
2	D’Ottavio et al. 2019 [11]	RCT	three	22	♂ + ♀	26 ± 3	22 ± 3	Advanced	Non-specific	5	11
3	Evangelista et al. 2019 [12]	RCT	three	58	♂ + ♀	27 ± 4	25	Hobby	Non-specific	5	10
4	Filipovic et al. 2016 [8]	RCT	two	22	♂	26 ± 3	24 ± 2	Professionals	Team sports	5	10
5	Filipovic et al. 2019 [13,14]	RCT	three	28/30	♂	23 ± 4	24 ± 2	Professionals	Team sports	6	11
6	Hussain et al. 2021/2022 [15,16]	RCT	three	60	♀	24 ± 2	22	Advanced	Team sports	5	9
7	Jawad et al. 2020 [17]	NRCT	two	10	♂	ng	ng	Professionals	Team sports	3	7
8	Kacoglu et al. 2021 [18]	RCT	two	38	♂ + ♀	22 ± 3	22 ± 2	Advanced	Non-specific	4	7
9	Ludwig et al. 2020 [19]	NRCT	two	30	♂	15–17	22	Advanced	Team sports	4	10
10	Martin-Simon et al. 2022 [20]	RCT	two	20	♂ + ♀	19–25	23	Advanced	Non-specific	4	7
11	Mathes et al. 2017 [21]	RCT	two	24	♂	23 ± 5	23	Advanced	Non-specific	5	10
12	Micke et al. 2022 [22]	RCT	two	18	♂	23 ± 3	22 ± 2	Advanced	Non-specific	5	11
13	Qin et al. 2022 [23]	RCT	two	20	♂	25 ± 4	24 ± 1	Hobby	Non-specific	6	10
14	Sadeghipour et al. 2021 [24]	RCT	three	30	♀	26 ± 2	23 ± 2	Hobby	Non-specific	5	7
15	Schuhbeck et al. 2019 [25]	RCOT	two	30	♂	28 ± 8	24	Advanced	Team sports	5	9
16	Wirtz et al. 2015/2016 [10,26]	RCT	two	20	♂	22 ± 2	24	Advanced	Non-specific	5	10
17	Zhang er al. 2021 [27]	RCT	two	10	♀	27 ± 4	22	Hobby	Strength	4	9
18	Zink-Rückel et al. 2021 [28]	RCT	two	54	♂	43 ± 14	27 ± 4	Hobby	Golf	8	14

^1^: If not specified, BMI was calculated based on body height and body mass (see BMI-data without SD); ng: not given; NCT: non-randomized controlled trial; RCT: randomized controlled trial; RCOT: randomized cross-over trial.

**Table 2:** Exercise characteristics reported by the eligible trials.

	Author	Study length [Months]	WB-EMS Mode	Comparable Volitional Exercise in Control Group?	EMS-Sessions n/Week × Length [min]	Exercise/WB-EMS Protocol Impulse Frequency (Hz), -Width (µs), -Duration (s), -Break (s), -Intensity (RPE) (In Addition to Sport-Specific Exercise)	Exercise/Activity in CG(s) (Without WB-EMS) (In Addition to Sport-Specific Exercise)	Adverse Effects Loss to FU (%)/ Attendance (%)/
1	Dörmann et al. [9]	1	SE	yes	2 × 20	DRT (same as CG) ⚡ by WB-EMS: 85 Hz, 350 µs, impulse during exercises, RPE ≥16 (CR20)	DRT: 4 exercises, 3 × 8–10 reps RPE ≥ 16 Power: 5 exercises., 3 × 5–10 reps/3 × 8 s	None/21/100
2	D‘Ottavio et al. [11]	1.5	SE	no	2 × 20	Ten isometric exercises ⚡ by WB-EMS: 350 µs, RPE 14–16: Two protocols: (a) 50 Hz, 4–6 s versus (b) 85 Hz, 4–4 s	DRT: 7 exercises, 3 × 10 reps 65% 1RM	None/0/100
3	Evangelista et al. [12]	2	SE	yes	2 × 20	DRT (same as CG) ⚡ by WB-EMS: 85 Hz, 350 µs, continuous impulse, RPE 7-8 (CR10)	DRT: 2 exercises 3 × 8–12 at RM	None/16/100
4	Filipovic et al. [8]	3.5	SE	yes	2 × 9	Squat jumps (same as CG) ⚡ by WB-EMS: 80 Hz, 350 µs, 4–10 s, up to RPE 18–19 (CR20)	Squat jumps: 3 × 10	None/0/100
5	Filipovic et al. [13,14]	2	SE	yes/ no ^1^	2 × 9	Squat jumps (same as CG) ⚡ by WB-EMS: 80 Hz, 350 µs, 4–10 s, RPE 16–19 (CR20)	(1) Squat jumps: 3 × 10 reps (2) Regular soccer routine only	None/4/100
6	Hussain et al. [15,16]	2	SE	yes/ no ^1^	3 × 20	Swing training + DRT (same as CG) ⚡ by WB-EMS: 85 Hz, 350 µs, 5–5 s, 50–80% maximum tolerable intensity	(1) Swing training (300 swings/week) + DRT 12 ex. 2–3 × 2–12 reps, 65–85% 1RM (2) Swing training only	None/0/100
7	Jawad et al. [17]	2	NSE	no	3 × 20	WB-EMS only: 85 Hz, 350 µs, continuous impulse, RPE 6–8 (CR10)	Rehabilitation program (19 DRT exercise, 3–4 × 10–20 reps) only	Not given
8	Kacoglu et al. [18]	1–1.5	SE	yes	2 × 25	DRT (same as CG) ⚡ by WB-EMS: 100 Hz, 400 µs, 5–10 s, RPE 8–9 (CR 10)	DRT: seated leg press (3 × 20 reps)	Not given
9	Ludwig et al. [19]	2.5	SE	yes	1 × 20	Strength/power training (same as CG) ⚡ by WB-EMS: 85 Hz, 350 µs, 4–4 s, RPE 6–7 (CR10)	20 min strength and power training (10 exercises)	None/0/97
10	Martín-Simón et al. [20]	1.5	SE	yes	1 × 13	3 sessions with 100–140 jumps with 1 session ⚡ by WB-EMS: 120 Hz, 350 µs, 5–10 s, max. tolerable intensity	3 sessions with 100-140 jumps each without WB-EMS	Not given
11	Mathes et al. [21]	1	SE	yes	3.5 × 60	Cycling (same as CG) ⚡ by WB-EMS: 80 Hz, 400 µs, 10–2 s, maximum tolerable intensity	Cycling at 60% peak power output	None/13/100
12	Micke et al. [22]	2	SE	yes	2 × ≈25	DRT (same as CG) ⚡ by WB-EMS: 85 Hz, 350 µs, adjusted to exercises 70% max. intensity	DRT: 5 exercises, 3x5-10 reps, RPE > 16 (CR20)	None/0/100
13	Qin et al. [23]	1.5	NSE	no	3 × 30	WB-EMS only: 85 Hz, 350 µs, 4–4s, RPE 6 (CR10) with easy exercises during the impulse phase	DRT: 5 exercises, 3–6 × 5 reps 80–100% 1RM	n.g./20/ng
14	Sadeghipour et al. [24]	1.5	NSE	no/ no ^1^	2 × 20	WB-EMS only: 85 Hz, 350 µs, 6–4 s, RPE 14–16 (CR20),	(1.) DRT: 4 ex., 3 × 8–12 reps, 60–80% 1RM (2.) Habitual exercise protocol only	Not given
15	Schuhbeck et al. [25]	3	SE	no	1 × 20	RT: (same as CG) ⚡ by WB-EMS: 85 Hz, 350 µs, 4–4s, ≥75% maximum intensity (+ habitual training routine)	6 weeks of static followed by 6 weeks of dynamic RT (+ habitual training)	None/13/100
16	Wirtz et al. [10,26]	1.5	SE	yes	2 × 10	Back squats (same as CG) ⚡ by WB-EMS: 85 Hz, 350 µs, 5s-1s at 70% max. tolerable intensity	Back squats: 4 × 10 reps to RM	None/0/100
17	Zhang et al. [27]	1.5	SE	yes	2 × 20–25	DRT: (same as CG) ⚡ by WB-EMS: 85 Hz, 350 µs, 60–100% WB-EMS device capacity	DRT: 4 exercises, 5 × to nRM at 85% 1RM	None/17/100
18	Zink-Rückel et al. [28]	4	NSE	no	1 × 20	WB-EMS only: 85 Hz, 350µs, 6s-4s, RPE 6-7 (CR10)	Habitual golf routine without WB-EMS	None ^2^/33/97

⚡: indicate “superimposed”; ^1^ studies with two control groups; ^2^ due to COVID-19 lock-down, eight participants quit the study, one participant was unable to attend the 16-week follow-up assessment; DRT: dynamic resistance exercise; n.g.: not given; nRM: non-repetition maximum; reps: repetitions; RPE: rate of perceived exertion; RT: resistance exercise; SE: superimposed exercise; NSE: non-superimposed exercise.

**Table 3:** Study outcomes reported in the eligible trials.

	Author	Study Endpoints
1	Dormann et al. [9]	(Isometric) Peak force (Fmax) and power (Pmax) leg press, leg extension, leg curl (leg flexion)
2	D’Ottavio et al. [11]	Force/velocity curves at 15, 35, 65, 85% 1 RM for squatting and bench press tests ^1^
3	Evangelista et al. [12]	1 RM test for back squat, biceps curls and “high pulley triceps extension exercise”
4	Filipovic et al. [8]	1 RM leg press
5	Filipovic et al. [13,14]	(Isometric) Peak force (Fmax) and power (Pmax) leg press, and leg curl (leg flexion)
6	Hussain et al. [15,16]	1 RM squat, bench press, trunk torsion via 3 RTF-test with prediction equation
7	Jawad et al. [17]	Maximum force of inner thigh muscles as determined by a hip adduction machine
8	Kacoglu et al. [18]	Maximum isokinetic force (torque) leg extension/flexion at 60°, 180°, 300°/s ^2^
9	Ludwig et al. [19]	(Isometric) Peak force (Fmax) knee flexion/extension, back flexion/extension, hip adduction/abduction
10	Martin-Simon et al. [20]	(Isometric) Peak force (Fmx) handgrip, peak power CMJ
11	Mathes et al. [21]	(Isometric) Peak force (Fmax) and power (Pmax) leg extension, leg curl (leg flexion)
12	Micke et al. [22]	(Isometric) Peak force (Fmax) and power (Pmax) leg press, leg extension, leg curl (leg flexion)
13	Qin et al. [23]	(Isometric) Peak force (Fmax) arm (elbow)-extensors and flexors,
14	Sadeghipour et al. [24]	1 RM leg press via 6 RTF-test with prediction equation
15	Schuhbeck et al. [25]	Maximum isokinetic force (torque) leg-extension/-flexion at 60°/s and 300°/s ^2^, peak power CMJ
16	Wirtz et al. [10,26]	(Isometric) Peak force (Fmax) leg press, -curl, abdominal press, back extension; power (Pmax) leg press, -curl
17	Zhang et al. [27]	(Software calculated) 1 RM and maximum velocity at 85% 1 RM for squat, bench press, deadlift, rowing exercise
18	Zink-Rückel et al. [28]	Maximum isokinetic force leg press at 0.5 m/s; (isometric) peak force (Fmax) trunk strength index ^3^

^1^ Force velocity curves for the 85% 1 RM tests were included in the analysis; ^2^ the test at 60° was included in the analysis; RTF: repetition to failure (test); ^3^ summary of six exercises (trunk extension, -flexion, lateral trunk flexion left/right side, trunk rotation left/right side).

## Data Availability

No new data were created or analyzed in this study.

## Abbreviations

The following abbreviations are used in this manuscript:

1 RM	One Repetition Maximum
CMJ	Counter Movement Jump
DRT	Dynamic Resistance Exercise Training
Fmax	Maximum Strength (Peak Force)
IVhet	Inverse Heterogeneity Model
nRM	Non-Repetition Maximum
PEDro	Physiotherapy Evidence Database
Pmax	Maximum Power (Peak Power)
RCOT	Randomized Cross-Over Trial
RCT	Randomized Controlled Trial
Reps	Repetitions
RPE	Rate of Perceived Exertion
RT	Resistance Exercise Training
RTF	Repetitions to fatigue
SD	Standard Deviation
SMD	Standardized Mean Differences
UKER	University Hospital Erlangen, Germany
TESTEX	Tool for the assEssment of Study qualiTy and reporting in Exercise
WB-EMS	Whole-Body Electromyostimulation

## Appendix A

**Table A1:** Underlying search strategies and their results [3].

Database	Search Date	Search Terms	Number of Hits
PubMed	21 February 2025	(WB-EMS OR “whole body electro myo stimulation” OR electromyostimulation OR “electrical muscle stimulation” OR electro-myo-stimulation OR electro-stimulation OR “integral electrical stimulation” OR “whole-body electrical muscle stimulation”) AND (athletic OR athlete OR sport OR performance OR trained)	1631
Cochrane	21 February 2025	(WB-EMS OR “whole body electro myo stimulation” OR elektromyostimulation OR “electrical muscle stimulation” OR electro-myo-stimulation OR electrostimulation OR “integral electrical stimulation” OR “whole-body electrical muscle stimulation”) AND (athletic OR athlete OR sport OR performance OR trained)	1332
CINAHL	21 February 2025	(WB-EMS OR “whole body electro myo stimulation” OR electromyostimulation OR “electrical muscle stimulation” OR electro-myo-stimulation OR electrostimulation OR “integral electrical stimulation” OR “whole-body electrical muscle stimulation”) AND (athletic OR athlete OR sport OR performance OR trained)	623
SPORTDiscus	21 February 2025	(WB-EMS OR “whole body electro myo stimulation” OR electromyostimulation OR “electrical muscle stimulation” OR electro-myo-stimulation OR electrostimulation OR “integral electrical stimulation” OR “whole-body electrical muscle stimulation”) AND (athletic OR athlete OR sport OR performance OR trained)	449
Web of science	21 February 2025	(WB-EMS OR “whole body electro myo stimulation” OR electromyostimulation OR “electrical muscle stimulation” OR electro-myo-stimulation” OR “whole-body electrical muscle stimulation”) AND (athletic OR athlete OR sport OR trained)	879
Other sources	21 February 2025	“WB-EMS, whole body electro myo stimulation, electromyostimulation, electrical muscle stimulation, electro-myo-stimulation, electrostimulation, whole-body electrical muscle stimulation” AND “athletic, athlete, sport, trained”	20
